# How do nursing home doctors involve patients and next of kin in end-of-life decisions? A qualitative study from Norway

**DOI:** 10.1186/s12910-016-0088-2

**Published:** 2016-01-14

**Authors:** Maria Romøren, Reidar Pedersen, Reidun Førde

**Affiliations:** Institute of Health and Society, Faculty of Medicine, University of Oslo, P.O box 1130, Blindern, 0318 Oslo, Norway

**Keywords:** Nursing home, End-of-life decisions, Life prolonging treatment, Advance care planning, Decision making capacity, Patient rights, Next of kin, Ethics, Justice

## Abstract

**Background:**

Ethically challenging critical events and decisions are common in nursing homes. This paper presents nursing home doctors’ descriptions of how they include the patient and next of kin in end-of-life decisions.

**Methods:**

We performed ten focus groups with 30 nursing home doctors. Advance care planning; aspects of decisions on life-prolonging treatment, and conflict with next of kin were subject to in-depth analysis and condensation.

**Results:**

The doctors described large variations in attitudes and practices in all aspects of end-of-life decisions. In conflict situations, many doctors were more concerned about the opinion of next of kin than ensuring the patient’s best interest.

**Conclusions:**

Many end-of-life decisions appear arbitrary or influenced by factors independent of the individual patient’s values and interests and are not based on systematic ethical reflections. To protect patient autonomy in nursing homes, stronger emphasis on legal and ethical knowledge among nursing home doctors is needed.

## Background

A substantial number of Norwegians spend their last days in nursing homes and approximately 45 % of all deaths occur here [1]. Nursing home residents are characterized by high age, frailty, chronic diseases and co-morbidity and deficits in activities of daily living [2, 3]. In a study from 2007, Selbaek, Kirkevold, and Engedal found that 60 % had moderate to severe cognitive impairment [4].

The incidence of critical events and critical decisions in nursing homes is high [5]. In Norway, health laws are launched to strengthen the patients’ autonomy, and in particular in end-of-life decisions [6]. In general, any kind of health care requires an informed consent, and the patient can decide whether relatives should be informed about his/her health situation. According to these laws, health care may be provided if it is deemed to be in the patient’s best interests and the patient gives or would have given permission to such care. When the patient lacks decision-making capacity, which is often the case when making end-of-life decisions in nursing homes [2, 7], the next of kin has a legal right to be informed about the patient’s medical condition. Further, the relatives should be asked to elicit information about the patient’s preferences. The treating medical doctor has the final say in treatment decisions. To know when it is in the patient’s best interests to limit life-prolonging treatment and replace it with palliative care is ethically challenging, in particular when the patient no longer is able to express an opinion. Systematically eliciting the patient’s values and interests in end-of-life care, is rarely a part of Norwegian nursing homes’ routines [8].

In 2009, a Norwegian county launched a program to teach personnel in 30 nursing homes technical competency to treat patients with intravenous (IV) hydration and antibiotics. In addition to the teaching of technical details, the nursing home staff received information about ethical and legal issues in end-of-life decisions. The program is being evaluated in order to find out whether the project affects mortality and morbidity of nursing home patients. Because this is a field in which value issues are salient, we found it important, as part of the evaluation, to explore how nursing homes deal with decisions to treat or not to treat actively. To our knowledge there exist few studies from nursing homes concerning these topics. This paper presents nursing home doctors’ descriptions of how the autonomy of the patient is taken into account in end-of-life decisions.

## Methods

We performed ten focus group interviews with 46 nursing home doctors in the period 2010–2011. All 57 nursing home doctors who were employed in the 30 nursing homes included in the described IV training course were invited to participate. In addition to the 46 who participated, another five were willing, but were not included because we, after the tenth interview, considered that the saturation point had been reached. Six doctors declined to participate.

### Sample

We interviewed 30 men and 16 women (age 26–66 and working experience one to 38 years). They were employed in 26 different nursing homes with 16 to 160 beds. The nursing homes had different types of services: rehabilitation, short-term and long-term care, palliative care and special units for patients with dementia. Some nursing homes had one type of services, whereas others had a combination.

Eight of the 46 doctors were employed in full time positions and two in half time positions. The other 36 were primary care physicians who worked 20 % in the nursing homes. The majority of these split their time into 40 % presence at the nursing home and 60 % availability for telephone consultations.

### Interviews

The interviews lasted around 60 min. After a brief introduction, the participants introduced themselves and shared their professional background and current situation. The participants were encouraged to share concrete, but anonymized, patient histories and examples that could illustrate their answers.

RP and RF were the main interviewers in five interviews each, while MR attended every interview as a co-interviewer and observer. We followed a semi-structured interview guide. Care was taken to involve all group participants in the discussions. The main topics included in the interview guide were: 1. How are decisions about intravenous treatment and other life-prolonging treatment made, including cooperation with nurses and other health personnel, routines for advance care planning or other involvement of the patient and the family, how does dementia affect the decision-making process? 2. How often are they in doubt about the right choice regarding active treatment? 3. How do they deal with conflicting opinions between patients/next of kin and/or health personnel?

### Analysis

We applied qualitative and manifest content analysis in our analysis of data [9]. After each interview the researchers discussed salient impressions from the interview, and a written summary was discussed by e-mail the following days. We aimed at a data-driven analysis; the interview notes were sporadically used later as a help to contextualize the transcripts. The interviews were tape recorded and transcribed verbatim. The written material was checked twice against the recordings. The unit of analysis and the content area for this article, was the interview text about how doctors involve patient and next of kin in end-of-life decisions. Our focus was the most explicit, visible, and obvious components of the text, i.e., the manifest content, rather than the latent or underlying meaning of the text [9].

The authors read all interviews individually and then discussed their interpretations. The interviews were reread several times with a focus on the content area, and relevant text was marked. The authors then discussed and agreed upon tentative main categories, made preliminary categorizations of the text, discussed uncertainties and adjusted the categories if necessary. The following categories were subject to in-depth analysis: 1) The doctors´ experiences with routine advance care planning or more informal end-of-life discussions in advance; 2a) Reasons for not involving the patient in end-of-life-decisions; 2b) Experiences with involving the patient; 3) Involvement of family. The interviews were reanalyzed using the developed categories to extract and sort all relevant text fragments or meaning units. The selected texts were condensed and typical quotes were selected. All authors discussed the analysis at various stages in the process.

### Ethical considerations

The Regional Committee for Medical Research Ethics approved the collaborative research project (reference no. 2009/1584a-1). Written informed consent was obtained from all of the participants. To protect the anonymity of the participants, any names and places in the transcribed text were replaced with numbers and characters.

## Results

We found major variations in the manner in which doctors reported their decisions to provide life-extending treatment, as well how they involve nursing home patients and their family members, both prior to, and in the relevant situations. Doctors gave different reasons for why they spoke with a patient and their family members. There were also different practices with regard to when these discussions took place, and with regard to who took the initiative for the discussion, whether only the patient participated, or only family members participated, or both, as well as the content of the discussions (Table 1).

**Table 1:** Involvement of patient and family members in decisions regarding treatment

Time of discussion • In conjunction with routine checks, and other similar situations^a^ • When the patient becomes noticeably more frail (anticipating the issue of life-extending treatment in the near future)^a^ • In conjunction with hospital admission • When the patient becomes acutely ill Form and content of discussions • Primarily information about regular routines, or if decisions have already been made a) Discussion in advance: “this is how things are usually done here” b) Discussion in an acute situation: inform of the decisions that have been made • Focus on dialogue • Allow family members to influence whether or not the patient should have treatment Doctors’ reasons for discussions • Considered routine practice • Family members have requested it • The doctor assesses the need for discussion or information • The nurses assess the need for discussion or information	

^a^Often defined as “advance care planning”

Both the type of department (somatic or dementia ward, short-term or long-term care), along with the doctor’s percentage of employment had an impact on the extent to which doctors became acquainted with patients and their family members, and involved them in decision-making processes. Many of the physicians who could only provide a few hours a week of supervision at the nursing homes described the nurses as the doctors’ “eyes and ears”. Major decisions regarding treatment could then be largely based on the nurse’s assessment of the patient’s health condition and quality of life.

### Doctors’ experiences with routine advance care planning

Generally, it was the physicians working full- or half time who reported having routine discussions with patients and family members regarding life-extending treatment. These took place shortly after admission to the nursing home, normally after 4–6 weeks. Questions surrounding these issues were often brought up again at the mid-year and yearly follow-ups.

The form and content of the advance care planning discussions were described in various ways, depending on whether the focus was on the patient or the family members, or whether the discussion was characterised by dialogue or one-way communication of information. Some reported that they asked direct questions, while others stated that they gradually worked their way up to the issue by gauging how strongly the family members and possibly also the patient chooses treatment when illness worsens. Many viewed these discussions as the start of a dialogue, where family members could prepare themselves for the patients’ death in the near future.

The concept “treatment clarification” was often used when referring to discussions with patients or family members with regard to life-extending treatment, but also in reference to discussions between doctors and nurses. For many, clarification of further treatment involved creating a shared plan for the scope of the life-extending treatment, while others defined it as a decision to refrain from giving active life-extending treatment. Several used these treatment discussions to curb family members’ expectations of treatment and hospital care. The doctors disagreed on appropriate topics to bring up with family members, and potentially also the patient, and had different views on the correct provision of treatment.

I think it’s not that easy to ensure treatment clarification for all possible events. And I do think it’s not a good idea to signalize that “here you can get whatever you want”, which I think may be hospital treatment. So if we involve the family members in this process, I’m afraid we’ll end up with even bigger problems…I mean, I’m not so sure that giving intravenous fluids to a nursing home patient is the right thing to do.

Most doctors considered hospital admittance an acceptable topic for discussion with their patients and family members, unlike the topic of cardiopulmonary resuscitation (CPR), which many justified by stating that CPR should never be performed on nursing home patients, regardless of the circumstances.

I should probably not say this, but it is tempting to just not ask, and say “This and that happened. Nothing was done.”

With respect to CPR – we had a discussion with the nurses where they asked what we would do, and then I gave them my opinion, saying that it was out of the question for all the elderly and all the dementia patients, that no one should be resuscitated. They should be allowed to die a natural death. If it happens, it will be out of the question to perform it on anyone here.

Those doctors who had had advance care planning discussions found them to be helpful. They had good experiences with the discussions and reported that they found it easier to bring up sensitive subjects when the patient was in a stable phase. They stated that family members are often grateful for the information and for being allowed the opportunity to be involved in decisions regarding treatment. This improved communication and reduced the risk of conflict with family members in acute circumstances.

As shown in Table 1, some of the doctors awaited the topic of life-extending treatment until the patient’s health condition became noticeably worse. It was not unusual for doctors to let the nurses decide when the time was right. If the doctors had these discussions after an acute serious illness had occurred, they spoke only with the family members. This meant that the doctors visited the nursing home outside working hours, spoke to the family members on the phone, or left the entire family discussion to the nurses. Many of them wanted better routines. The lack of advance care planning may have resulted in increased uncertainty among the staff, as well as conflicts with family members, and was described as unnecessarily demanding. It may have also resulted in the implementation of treatment that should never have been provided.

### Why are patients not involved?

In each of the interviews, more attention appears to be given to involving family members than to involving the patient, and the doctors had significantly more focus on eliciting the family’s wishes than the wishes of the patient. Doctors who argue against involving the patient and even the family on the issue of life-extending treatment had a number of various reasons for this, such as the uncertainty of what could occur, or that the patient or family members might change their mind at some point.

Many of the doctors said that talking with patients about life-extending treatment was too time-consuming, and that practical problems made it difficult to give this issue priority. Trouble with finding a suitable time for such a discussion was one argument doctors from both short-term and long-term wards had against advance care planning. Some said they had thought of having such discussions, but that they did not have the time, nor did they know how to carry them out.

…no one really seems to know what to do, I think. Many of the discussions we should have had are just not possible to carry out, and now our department manager is telling me to spend my time on documentation and filling in diagnoses in the medical journal system rather than see the patients, because the top management is breathing down our necks, and it’s terribly difficult. We have had a few discussions lately, but with so many patients there just isn’t enough time for it.

The doctors felt that there was little reason to discuss treatment with dementia patients, either prior to, or in the midst of an acute illness. “Dementia” was, however, often used as a general term, with little distinction, and rarely problematized. But even when patients are lucid, many doctors feel it is either inappropriate or too difficult to discuss issues regarding treatment directly with the patient because they do not wish to worry the patient. Several argued against asking patients about their thoughts and wishes prior to the onset of serious illness because they wanted to shield the patients from the subject of illness and death. Some also believed that doctors should be cautious about asking patients about their wishes in the event of acute illness, because the patient may think differently at that time than when they are in their habitual state. Several of them role-played a patient discussion in order to illustrate why it would be better to shield the patient from this subject.

They’re lying there, right, and we stand over them and ask them if they want to be saved if they become seriously ill. And I have to say that most times I just don’t bring up, right? Because then they’ll get the treatment they, uh, should have, when they become seriously ill. I mean, instead of making that decision themselves, it’s…right? I think it’s a difficult question, and maybe they shouldn’t have to answer it.

Some of the doctors stated that the ideal of self-determination is illusory for elderly patients who have been taught to do as the doctor says, and that some patients would rather the doctor spoke to their families than to them.

Many doctors do not involve patients because they themselves had strong opinions on what quality of life entails for patients, and what is in their best interests. Life-extending treatment may be limited because staff interprets patients’ refusal of medicine, food and drink, as a form of resignation, where the patient, having lived a long life, is “full of years”.

Some doctors admitted that they avoided advance care planning discussions because they found them difficult.

I think the major obstacle lies with us. Because I believe that the elderly, at least those who, in a way, are still cognisant and have not become completely senile, are far more able to talk about these things than we think. We’re the ones who are afraid. You tend to shove it aside, because it’s uncomfortable, too difficult. I think we need to have some sort of relationship with our patients before we start talking about these kinds of things…we really should.

### When patients are involved

A minority of doctors attempted to include the patients in decisions regarding treatment and level of treatment – some of them early in the process, and others during the actual acute illness situation. Doctors who speak with their patients state that they adjust the dialogue to the patients’ level of function and report almost no negative feedback. Some doctors speak with their patients in order to assess their preferences, for instance, if they would want to be admitted to hospital. Others, however, appear to place clear constraints on the discussion, promoting their own stances on the issue.

And if I speak with the patient and say that “if you have a heart attack”, this is if the patients are 83, 84 and over 90. Uh, …“what do you want us to do? Save you or try to save you and do CPR and call an ambulance and…” And many of them say “No, I don’t want that. I want to end my life if that happens.”

No one reported pressure, disagreements or conflicts with the patients. Doctors stated that most of the patients who were asked replied that they did not want life-extending treatment. But in some cases the patient surprised them by expressing a wish for treatment, even when the doctor did not believe this would be appropriate or beneficial, or they thought they knew what the patient wanted. In these cases the doctors allowed the patients to decide. The following quote illustrates how both family members and doctors may be wrong about a patient’s wishes.

A man chose to begin treatment with IV antibiotics and fluids, and he improved. And then we had a kind of network meeting with the family afterwards, and they said that “if Dad could decide, he would almost certainly not want to begin any kind of treatment.” And I thought that was a good place to start. So we talked about what we would do the next time he became ill again, because that might happen soon. So then we stopped the meeting and I said, “Can I ask him?” And they said yes, they thought I should. So I went in and spent a good deal of time talking with him. I was sure he would say that the next time it happened he wouldn’t want treatment, because he remembered how it had been, that he was very ill…and close to death…and then we would have his wishes on this matter. But it was just the opposite. No, he remembered it, and was grateful for it, and he wanted to continue living, and wanted to have treatment the next time as well.

### Family members as decision makers?

The doctor primarily has contact with the family members with regard to life-extending treatment for the patient, both prior to and during the patient’s acute illness. Doctors often used the term “family conversations” in referring to discussions regarding life-extending treatment, and often described family members as a resource. Although it was more or less taken for granted that all families of patients would want to be involved, there were a few examples of family members who did not wish to be included in discussions concerning treatment, and who were perhaps worried about the responsibility associated with these decisions. Many of them made it clear that it was the doctor who should make the final decisions regarding treatment to avoid making the family feel they had too much responsibility.

Attitudes regarding confidentiality in relation to family members varied. Some doctors stated that they asked the patient for permission to involve family members, but many believed it was natural to have an open dialogue with the families of all patients at the nursing home, without having to ask the patient for permission.

I think the families would start to wonder if they phoned me and I said “I have to go and ask if I’m allowed to speak with you” first…

Dialogue and exchange of information with the family took place regardless of whether the patient was competent to give consent. Few carried out systematic assessments of patients’ competence to consent. Several of the doctors, especially the supervising physician, did not consider such assessments to be of any practical use, and based their opinions of competence to consent on their own judgement or intuition.

There are major differences in the roles doctors allow family members to have with regard to decision-making. At one extreme, family members were used to help the doctor better understand the patient’s wishes, and at the other extreme, family members were allowed to decide whether or not to provide treatment, in some cases also against the expressed wishes of the patient.

Yes, but, I mean…My prestige doesn’t mean as much as letting the family have their way. The family is always right, so if they insist on hospital admission, we’re always able to manage that.

*Interviewer: But what if that’s not what the patient wants?”*

Yeah, true, the patient is being manipulated then. He has to be manipulated by his family members in one way. And…It does often happen that they’re talked into things by their family members, in a way.

There were differences in the frequency with which doctors experienced disagreements with family members regarding life-extending treatment, and to what extent they allowed the family to be the true decision-makers. Many of them made the conscious choice to facilitate opportunities for families to speak with them whenever needed, in order to prevent mistrust or disagreements. Communication, in the event of a disagreement, was described as taking various forms, from “listening to the family” and “understanding one another” to “trying to convince the family” and “get them to agree”. Most conflicts dealt with family members who wished for a more active treatment than the patient wanted, or that the the doctor believed was in the patient’s best interests.

Although they rarely yielded to pressure by the family, they did state that they would give in if a conflict came to a standstill. Several of them emphasised the necessity and benefits of dialogue with family members, but stated also that these discussions could be time-consuming. In examples provided by the doctors, the doctors appeared certain that the patient had refused treatment – even when the patient’s wishes were not known. Very few of them expressed any doubts about life-extending treatment, or whether there might have been other correct alternative courses of action, or whether they might have been wrong.

Doctors defended having to bow to pressure, stating that it was “ethically unproblematic”, “not directly wrong” or “more or less okay” and they did not problematize the fact that they gave the patient treatment which the patient did not want, or that they themselves believed was not in the patient’s best interests.

I mean, if you end up with these sort of ethical issues of whether or not to start intravenous treatment, then…you’re not exactly doing anything wrong in terms of starting…You need to be pretty sure of yourself to not begin treatment sometimes, and that can lead to some very strong criticism from the family if there’s no agreement ahead of time.

Conflicts were described as both unpleasant and demanding. Worries about ending up on the front page of the newspaper or having complaints lodged against them to the County Governor’s office were also major reasons for allowing family members to decide.

I have, on a number of occasions, treated cancer patients for a much longer period of time than I would have wanted had the patient been a member of my family, so you could say that this happened because of pressure from the families. Yes, I could have said “no, that’s not going to happen”, but on the other hand, when people say they wouldn’t be able to live with themselves unless all options have been tried, that’s a pretty heavy statement. And they are after all very close relatives. I can live with having over-treated a patient who still dies after a relatively short period of time…

A few doctors stated that they would never bow to pressure from family members. They said that they maintained focus on the patient’s best interests and were certain that their opinions on what was best for the patient were correct. The same doctors also provided examples of complaints lodged against them to the County Governor by family members, or having been reported in the media.

## Discussion

This study reveals variation among nursing home doctors in how they involve patients and next of kin in end-of-life decisions. Some of the variations can be explained by the health condition of the patient. However, many important decisions appear arbitrary or influenced by factors independent of the patient, or strongly influenced by individual values; such as the doctor’s assessment of the patient’s quality of life which is also is a value assessment, the next of kin’s own opinion of what should be done, the individual doctor’s attitudes towards life-prolonging treatment to nursing home patients or to patient autonomy in general. In consequence, these serious decisions may be influenced by coincidences rather than based on systematic ethical reflections adapted to the individual patient’s conditions, values, and interests.

Norwegian law and guidelines emphasize patients’ right to be included in end-of-life decisions, and to refuse life-prolonging treatment [6]. These regulations are violated if doctors neglect the patient’s values and opinions, or if the next of kin are given greater decision-making authority than they should have, sometimes also leading to interventions which are contrary to the patient’s will and best interests. According to the law, a competent patient should give consent before the professionals give relatives medical information. In this study, the nursing home doctors describe that next of kin are consulted and informed automatically, also in cases when the patient still is competent. Furthermore, the patient’s competence to consent is often not assessed. A recent Norwegian study among geriatric patients reveal that not all elderly want their relatives to have medical information or to be consulted about treatment options [10].

In our study, the doctors seem to have a strong focus on the next of kin when end-of-life decisions are being made. Although the patients’ families sometimes could challenge the doctors’ clinical judgment, most doctors looked upon the families as a resource and underlined the importance of communication with the next of kin to build trust and a common understanding of the best thing to do. Dreyer, Forde and Nortvedt (2009) found that if next of kin were not involved in end-of-life planning before the nursing home patient became acutely ill, conflicts and distrust in the decisions taken by the doctor developed more easily, sometimes leading to overtreatment [11]. Thus, improved routines for involvement of patient and next of kin, which may appear time consuming, may actually save time, energy and resources, prevent conflicts, and lead to decisions which are in line with the patient’s values and preferences. This was also the experience of the doctors in our study.

### The art of involving the patient in end-of- life decisions

Some of the doctors did not involve the patients in advance care planning because they felt that it would upset them. However, the few doctors who did this as part of their routines described that they always had found this to be a good thing to do. If resistance against direct involvement of patients and next of kin is related to the doctor’s own feelings of uneasiness, this calls for communication education in nursing homes.

Some of the doctors described episodes where they felt they knew the patient’s preferences, but could be surprised when the patient was involved in the decision, the patient had changed his or her mind about life-prolonging treatment. This underlines that involvement of patients through advance care planning is not a one-time effort, but a continuous process. In addition, we know that not all patients and not all next of kin want to have a say in end-of-life decisions [12, 13]. Therefore involvement must start with a first approach: to which extent do they want to be involved in end-of-life decisions? It is part of a nursing home doctor’s skills to adapt their approach to the individual patient’s needs.

Although these doctors had received information about relevant laws and ethical guidelines in connection with the IV training course, we found substantial variations in the doctors’ knowledge of laws and ethical guidelines. This together with the great individual variations in how the doctors involve patients and next of kin underlines a great need for education and supervision in ethics, law and communication for nursing home doctors. It is not acceptable that coincidences decide whether patients are met with treatment nihilism or are submitted to treatment which the patient or the doctor feel is not for the best of the patient.

The fact that so few of our interviewees describe that their decisions are taken under uncertainty may also indicate a need for such education. Several of the doctors interviewed also expressed that they wished to develop improved routines and wished to have more time to take this important part of their work seriously.

### Study weaknesses

This study is based on interviews with doctors from one (albeit big) county in Norway. Our findings are based on how the doctors describe their practice. An observation study would have given a better picture of how they actually involve patients and next of kin. In addition, doctors may be “eager to please” when normative issues are discussed among colleagues.

We have not identified studies directly comparable with ours. However, patients’ unmet need for information, and the problems and complexity in end-of-life communication, are shown in different contexts for care across different countries and health care systems [14–17].

## Conclusions

This study reveals variation among nursing home doctors in how they involve patients and next of kin in end-of-life decisions, and many important decisions appear arbitrary and not based on systematic ethical reflections. Not uncommonly, the next of kin are given greater decision-making authority than they should have, sometimes also leading to interventions which are contrary to the patient’s will and best interests. The few doctors who involve the patients in advance care planning as part of their routines described that they always had found this to be a good thing to do.

The results underline a great need for education and supervision in ethics, law and communication for nursing home doctors, to improve the decision-making processes and avoid large and haphazard variations in practice. Improved routines for involvement of patient and next of kin may save time, energy and resources, prevent conflicts, and lead to decisions in line with the patient’s values and preferences. It is not acceptable that coincidences decide whether patients are met with treatment nihilism or are provided treatment that the patient or the doctor feel is not for the best of the patient.

## Abbreviations

CPR: cardiopulmonary resuscitation
IV: intravenous
